# Postprandial Plasma Glucose between 4 and 7.9 h May Be a Potential Diagnostic Marker for Diabetes

**DOI:** 10.3390/biomedicines12061313

**Published:** 2024-06-13

**Authors:** Yutang Wang, Yan Fang, Christopher L. Aberson, Fadi J. Charchar, Antonio Ceriello

**Affiliations:** 1 Discipline of Life Science, Institute of Innovation, Science and Sustainability, Federation University Australia, Ballarat, VIC 3350, Australia; 2 Department of Psychology, Humboldt State University, Arcata, CA 91711, USA; 3 RCCS MultiMedica, Via Gaudenzio Fantoli, 16/15, 20138 Milan, Italy; antonio.ceriello@hotmail.it

**Keywords:** postprandial, non-fasting, fasting, glucose, diabetes

## Abstract

Postprandial glucose levels between 4 and 7.9 h (PPG_4–7.9h_) correlate with mortality from various diseases, including hypertension, diabetes, cardiovascular disease, and cancer. This study aimed to assess if predicted PPG_4–7.9h_ could diagnose diabetes. Two groups of participants were involved: Group 1 (4420 participants) had actual PPG_4–7.9h_, while Group 2 (8422 participants) lacked this measure but had all the diabetes diagnostic measures. Group 1 underwent multiple linear regression to predict PPG_4–7.9h_ using 30 predictors, achieving accuracy within 11.1 mg/dL in 80% of the participants. Group 2 had PPG_4–7.9h_ predicted using this model. A receiver operating characteristic curve analysis showed that predicted PPG_4–7.9h_ could diagnose diabetes with an accuracy of 87.3% in Group 2, with a sensitivity of 75.1% and specificity of 84.1% at the optimal cutoff of 102.5 mg/dL. A simulation on 10,000 random samples from Group 2 revealed that 175 participants may be needed to investigate PPG_4–7.9h_ as a diabetes diagnostic marker with a power of at least 80%. In conclusion, predicted PPG_4–7.9h_ appears to be a promising diagnostic indicator for diabetes. Future studies seeking to ascertain its definitive diagnostic value might require a minimum sample size of 175 participants.

## 1. Introduction

According to the World Health Organization, an estimated 422 million individuals globally grapple with diabetes [1], a condition linked to severe complications such as heart disease, chronic kidney disease, and blindness [2], culminating in 1.5 million annual deaths [1]. The direct health expenditure for diabetes worldwide reached approximately USD 760 billion in 2019, with projections soaring to around USD 825 billion by 2030 [3]. Surprisingly, in 2021, nearly half of diabetic adults remained undiagnosed, constituting approximately 239.7 million individuals [4]. Consequently, prioritizing research endeavors aimed at enhancing diabetes detection, pinpointing risk factors, and developing therapies is paramount in clinical practice [5].

Postprandial plasma glucose has long been recognized to play a vital role in diabetes-associated complications [6,7,8] and glycemic control [9,10,11]. Its positive correlation with cardiovascular disease incidence [12,13,14,15] and mortality [16], cancer mortality [17,18], and all-cause mortality [12,13,14,17,19] underscores its potential as a therapeutic target to mitigate diabetes-associated morbidity and mortality [7]. However, conventional assessments typically focus on early postprandial glucose levels, such as those measured at 1 h [12] or 2 h after a meal [13,14,15,17,18,19]. Yet, the susceptibility of these measurements to dietary variations and timing discrepancies poses challenges, potentially skewing results [15,16,17,20].

Recent findings highlight the stability and significance of postprandial glucose levels measured between 4 and 7.9 h after a meal (PPG_4–7.9h_) [21,22]. Hourly PPG_4–7.9h_ levels were similar across the duration from 4 to 7.9 h [21,22]. Plasma glucose returned to baseline four hours after a meal regardless of the type of the meal (normal or high carbohydrate) or the time of the meal (breakfast, lunch, and dinner) in healthy individuals [20]. These results suggest that the interval of 4 to 7.9 h after a meal may reflect glucose homeostasis irrespective of meal composition or timing, offering a promising window for assessment. Moreover, PPG_4–7.9h_ exhibits positive associations with mortality from prevalent conditions like hypertension, cardiovascular disease, and cancer [21,22], further emphasizing its clinical relevance.

Diabetes diagnosis relies on fasting plasma glucose, 2 h plasma glucose during OGTT, or HbA_1c_ [23]. However, there are some limitations to using these tests. Fasting plasma glucose and OGTT require fasting and, thus, pose practical challenges. Fasting can be inconvenient and even risky, particularly for vulnerable individuals who may experience hypoglycemia while waiting for blood collection [24]. The HbA_1c_ test does not require fasting and minimizing daily fluctuations due to lifestyle changes; however, this test’s diagnostic accuracy is compromised. For instance, factors such as hemodialysis, HIV treatment, age, ethnicity, pregnancy, and hemoglobinopathies can influence HbA_1c_ readings, leading to potential misdiagnoses [23]. The American Diabetes Association recommends prioritizing fasting plasma glucose and 2 h plasma glucose if discrepancies arise between HbA_1c_ and glucose values [23]. In addition, HbA_1c_ has poor sensitivity in diabetes diagnosis and misses a large proportion of diabetes that is detected by OGTT [25], the gold standard method for diabetes diagnosis [26,27]. For example, the proportion of OGTT-diagnosed diabetes that HbA_1c_ can detect was reported to be 43% in Denmark, 25% in the UK, 17% in Australia, 30% in Greenland, 20% in Kenya, 78% in India [28], and 30% in China [29]. Moreover, the HbA_1c_ test is more expensive than the glucose test [30,31]. Therefore, there remains a need to explore additional diabetic diagnostic tools that provide diagnostic accuracy while upholding convenience and safety, alongside the capacity to forecast clinical outcomes.

Given the potential diagnostic utility of PPG_4–7.9h_, exploring its feasibility in diagnosing diabetes warrants attention. However, existing datasets lack concurrent measurements of PPG_4–7.9h_ alongside traditional diagnostic indicators for diabetes. This gap impedes the accurate estimation of the sample size necessary for investigating the diagnostic efficacy of PPG_4–7.9h_.

To address this, the present study leveraged the comprehensive National Health and Nutrition Examination Survey (NHANES) dataset in which a large number of predictors of PPG_4–7.9h_ are available. This study aimed to construct a model predicting PPG_4–7.9h_ in one group (Group 1, *n* = 4420) of participants who had actual PPG_4–7.9h_ values; subsequently, PPG_4–7.9h_ values were estimated using this predictive model in another group (Group 2, *n* = 8422) of participants who lacked PPG_4–7.9h_ but had complete diabetes diagnostic profiles, i.e., fasting plasma glucose, 2 h plasma glucose during oral glucose tolerance test (OGTT), and hemoglobin A_1c_ (HbA_1c_) [23,26]. The diagnostic suitability of predicted PPG_4–7.9h_ for diabetes was then investigated in Group 2 participants, and the sample size that would be required by future studies aiming to investigate the true diagnostic value of PPG_4–7.9h_ for diabetes was estimated. Therefore, this study aimed to investigate the diagnostic potential of predicted PPG_4–7.9h_ for diabetes, which may lay the groundwork for future investigations and clinical applications.

Antidiabetic medications have confounding effects on blood glucose levels [32,33,34]. Therefore, this study excluded those who were taking antidiabetic medications or with unknown medication status.

## 2. Materials and Methods

### 2.1. Study Participants

This study included adult participants (aged ≥20 years) from NHANES III (1988–1994) and the subsequent eight cycles of NHANES from 1999 to 2014 [35]. Two groups of participants were selected from the NHANES participants: Group 1 (the postprandial group) and Group 2 (the fasting group).

**Figure 1:**
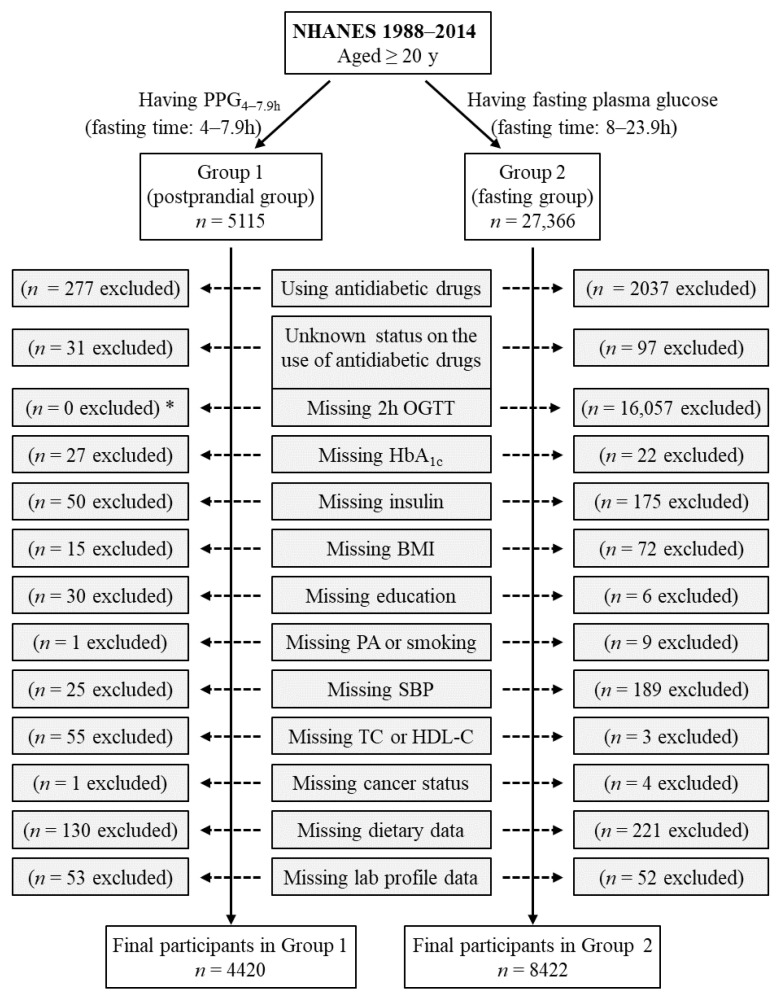
Flow diagram of the study participants. Group 1 participants had postprandial plasma glucose measured from blood taken between 4 and 7.9 h (PPG_4–7.9h_). Group 2 participants had fasting plasma glucose with a fasting time between 8 and 23.9 h. *: participants in Group 1 did not have OGTT data, and OGTT was not an exclusion criterion for Group 1 participants. BMI, body mass index; HbA_1c_, hemoglobin A_1c_; HDL-C, high-density lipoprotein cholesterol; NHANES, National Health and Nutrition Examination Survey; OGTT, oral glucose tolerance test; PA, physical activity; SBP, systolic blood pressure; TC, total cholesterol.

Group 1 included all participants who had postprandial plasma glucose measured from blood taken between 4 and 7.9 h (PPG_4–7.9h_, *n* = 5115). Participants using antidiabetic drugs (*n* = 277) or with unknown status on the use of antidiabetic drugs (*n* = 31) were subsequently excluded. Individuals who had missing data from the following variables were also excluded: HbA_1c_ (*n* = 27), insulin (*n* = 50), body mass index (*n* = 15), education (*n* = 30), smoking (*n* = 1), systolic blood pressure (*n* = 25), total cholesterol (*n* = 25), high-density lipoprotein (HDL) cholesterol (*n* = 30), cancer (*n* = 1), dietary intake data (carbohydrate, protein, fat, and total energy, *n* = 130), laboratory profile (*n* = 53 including *n* = 50 for potassium, *n* = 1 for total protein, and *n* = 2 for bilirubin). Therefore, the remaining 4420 participants were included in the final analysis for Group 1 (Figure 1).

Group 2 included those who had fasting plasma glucose (fasting time of 8–23.9 h), *n* = 27,366. Participants using antidiabetic drugs (*n* = 2037) or with unknown status on the use of antidiabetic drugs (*n* = 97) were subsequently excluded. Individuals who had missing data from the following variables were also excluded: 2 h plasma glucose during OGTT (*n* = 16,057), HbA_1c_ (*n* = 22), insulin (*n* = 175), body mass index (*n* = 72), education (*n* = 6), physical activity (*n* = 2), smoking (*n* = 7), systolic blood pressure (*n* = 189), total cholesterol (*n* = 3), cancer (*n* = 4), dietary intake data (carbohydrate, protein, fat, and total energy, *n* = 221), and laboratory profile (*n* = 52 including *n* = 12 for potassium, *n* = 12 for calcium, *n* = 1 for phosphorus, *n* = 14 for bicarbonate, *n* = 7 for total protein, and *n* = 6 for bilirubin). Therefore, the remaining 8422 participants were included in the final analysis for Group 2 (Figure 1). Group 2 participants had all three diabetes diagnostic measures, namely, fasting plasma glucose, 2 h plasma glucose during OGTT, and HbA_1c_.

### 2.2. Diabetes Definition

Diabetes was diagnosed based on criteria established by the American Diabetes Association [23,36], which included a fasting plasma glucose level equal to or exceeding 126 mg/dL, a 2 h plasma glucose during OGTT equal to or exceeding 200 mg/dL, or an HbA_1c_ level in whole blood equal to or exceeding 6.5%.

### 2.3. PPG_4–7.9h_

Blood was drawn from participants. The time of blood collection and last caloric intake were recorded, and the fasting time was calculated. Blood was taken between 4 and 7.9 h after the last caloric intake was used to measure PPG_4–7.9h_ by the hexokinase-mediated reaction method as previously described [37].

### 2.4. Potential PPG_4–7.9h_ Predictors

The following variables were retrieved from the NHANES data set and treated as potential factors for PPG_4–7.9h_ as they may affect plasma glucose levels: age, sex, ethnicity, body mass index, education, income, physical activity, smoking, alcohol intake, dietary intake (carbohydrate, protein, fat, and total calorie), systolic blood pressure, total cholesterol, HDL cholesterol, family history of diabetes, cancer diagnosis, use of antihypertensive medication, use of cholesterol-lowering medication, circulating ionic profile (potassium, calcium, sodium, phosphorus, bicarbonate, and chloride), circulating enzymatic and metabolic profile (alanine aminotransferase, aspartate aminotransferase, bilirubin, blood urea nitrogen, creatinine, and uric acid), serum protein, serum albumin, serum insulin, HbA_1c_, and fasting time.

### 2.5. Statistical Analyses

The participants’ baseline characteristics were described using numbers with percentages for categorical variables, median with interquartile range for non-normally distributed continuous variables [38], and mean with standard deviation (SD) for normally distributed continuous variables in the presented data [39].

The associations of PPG_4–7.9h_ with potential predictors were analyzed using simple linear regression [40]. The significant predictors, determined by the simple linear regression, were then added to the multiple linear regression model to predict PPG_4–7.9h_ [41].

The following variables were natural log transformed to improve data distribution prior to linear regression [42]: PPG_4–7.9h_, fasting plasma glucose, 2 h plasma glucose during OGTT, body mass index, systolic blood pressure, total cholesterol, HDL cholesterol, dietary carbohydrate intake, dietary protein intake, dietary fat intake, dietary caloric intake, alanine aminotransferase, aspartate aminotransferase, bilirubin, blood urea nitrogen, serum creatinine, serum insulin, and blood HbA_1c_.

The performance of predicted PPG_4–7.9h_ for classifying diabetes was assessed by receiver operating characteristic (ROC) curve analysis [43,44]. The optimal cutoff of predicted PPG4–7.9h was determined by the Youden Index [45].

Power estimation was carried out through simulations involving 10,000 randomly generated samples with various sample sizes derived from the pool of 8422 participants in Group 2 [46,47]. Within each sample, the diagnostic accuracy, sensitivity, and specificity of predicted PPG_4–7.9h_ for diabetes diagnosis were computed using the following formulas [48,49,50]:

Diagnosis accuracy = (number of participants correctly diagnosed with diabetes + number of participants correctly diagnosed without diabetes)/total number of participants in the sample.

Sensitivity = number of participants correctly diagnosed with diabetes/total number of participants with actual diabetes.

Specificity = number of participants correctly diagnosed without diabetes/total number of participants without actual diabetes.

Among the 10,000 random samples, the percentage exhibiting a diagnostic accuracy of 80%, which is deemed a minimum threshold for an excellent diagnostic marker [51], was computed to determine the diagnostic power of PPG_4–7.9h_ in identifying diabetes. Mean sensitivity and specificity values were calculated from the 10,000 samples, and their 95% confidence intervals were derived from the 2.5th and 97.5th percentiles of the sensitivity and specificity readings [52]. Furthermore, an investigation into a diagnostic accuracy of 81% was conducted to assess power and sample size requirements.

The null hypothesis was rejected for two-sided values of *p* < 0.05. Power and sample size were estimated using the R program, and all other analyses were performed using SPSS version 27.0 (IBM SPSS Statistics for Windows, Armonk, NY, USA, IBM Corporation).

## 3. Results

### 3.1. Baseline Characteristics

Group 1 (the postprandial group) included 4420 participants with a mean (SD) age of 49 (19) years, and Group 2 (the fasting group) had 8842 participants with a mean (SD) age of 48 (17) years (Table 1). All other characteristics of the participants are described in Table 1.

### 3.2. Factors Associated with PPG_4–7.9h_ in Group 1 of 4420 Participants, Assessed by Simple Linear Regression

Simple linear regression analysis identified 30 factors associated with PPG_4–7.9h_ (Table 2). These factors included age, sex, ethnicity, body mass index, education, income, physical activity, smoking, alcohol intake, dietary carbohydrate intake, dietary fat intake, dietary caloric intake, systolic blood pressure, total cholesterol, HDL cholesterol, cancer diagnosis, use of antihypertensive medications, and certain circulating biomarkers. These biomarkers included potassium, calcium, phosphorus, bicarbonate, chloride, alanine aminotransferase, aspartate aminotransferase, bilirubin, blood urea nitrogen, creatinine, uric acid, insulin, and HbA_1c_.

Simple linear regression showed that the following seven factors were not associated with PPG_4–7.9h_: family history of diabetes, use of cholesterol-lowering medications, dietary protein intake, serum sodium, serum protein, serum albumin, and fasting time (Table 2).

### 3.3. Predictive Model for PPG_4–7.9h_ Using Multiple Linear Regression in Group 1 of 4420 Participants

The predictive model was constructed using multiple linear regression (Table 3). The predictors were the 30 factors that were identified as significantly associated with PPG_4–7.9h_ in simple linear regression (Table 2). These 30 predictors accounted for 42.9% of the variation in PPG_4–7.9h_ (R square, Model 7, Table 3). The individual coefficients for each predictor in the final model (Model 7, Table 3) are listed in Table 4.

In Group 1, the predicted PPG_4–7.9h_ values were generated utilizing the predictive model comprising 30 predictors, along with their respective coefficients listed in Table 4. To assess the model’s performance, the difference between the predicted and actual PPG_4–7.9h_ values was calculated. Analysis revealed that approximately 80% of participants exhibited predicted PPG_4–7.9h_ values within a margin of 11.1 mg/dL from the actual values (Table 5). These findings indicated that the predictive model demonstrated a commendable level of accuracy.

### 3.4. Predicted PPG_4–7.9h_ for Diabetes Diagnosis in Group 2 of 8422 Participants

**Figure 2:**
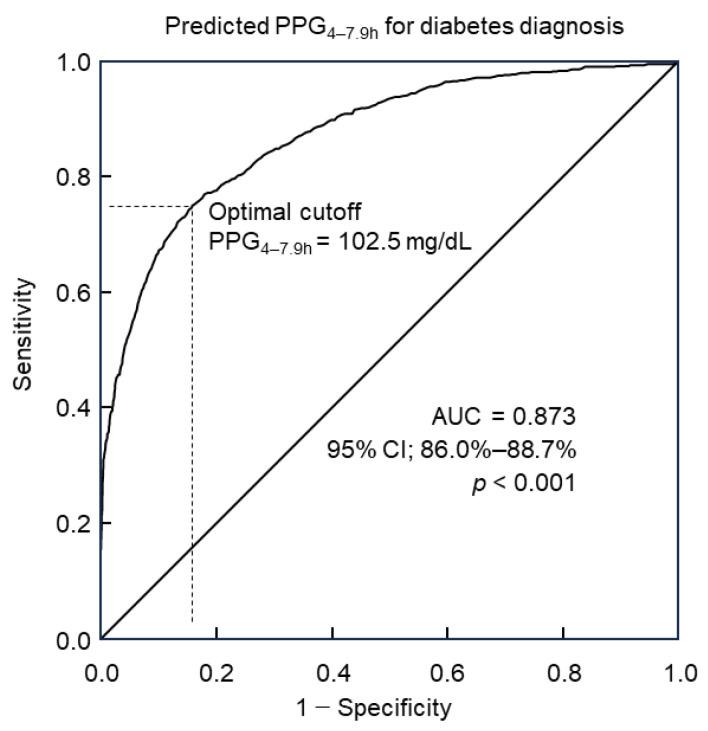
ROC curve analysis of predicted PPG_4–7.9h_ for diabetes diagnosis. The optimal cutoff was 102.5 mg/dL, with a sensitivity of 75.1% and specificity of 84.1%. The area under the curve (AUC) = 0.873. CI, confidence interval; PPG_4–7.9h_, postprandial plasma glucose measured from blood taken between 4 and 7.9 h; ROC, receiver operating characteristic.

Predicted PPG_4–7.9h_ values were computed for Group 2 of 8422 participants utilizing the predictive model incorporating 30 predictors along with their corresponding coefficients (Table 4). Diabetes diagnosis followed the diagnostic criteria outlined by the American Diabetes Association. The utility of predicted PPG_4–7.9h_ in diagnosing diabetes was analyzed through ROC curve analysis. Results revealed that predicted PPG_4–7.9h_ could discern diabetes with an accuracy of 87.3% (95% confidence interval: 86.0%–88.7%), as indicated by the area under the curve (AUC, Figure 2). Further analysis via the Youden index indicated that the optimal cutoff point of predicted PPG_4–7.9h_ for diabetes diagnosis was 102.5 mg/dL. This threshold was associated with a diagnostic sensitivity of 75.1% and specificity of 84.1% (Figure 2).

### 3.5. Power and Sample Size Estimation for Predicted PPG_4–7.9h_ to Diagnose Diabetes in Group 2 of 8422 Participants

Power analysis for diagnosing diabetes using predicted PPG_4–7.9h_ was conducted in Group 2 through the simulation of 10,000 random samples, each with varying sample sizes ranging from 50 to 300 participants. Diabetes prediction was defined as a predicted PPG_4–7.9h_ equal to or above the optimal cutoff of ≥102.5 mg/dL (Figure 2), and actual diabetes status was determined based on the criteria outlined by the American Diabetes Association. The accuracy of predicted diagnoses for each of the 10,000 random samples was assessed by comparing them with the actual diabetes status.

In evaluating the accuracy, it is notable that an accuracy falling within the range of 0.8 to 0.9 is considered excellent, while an accuracy between 0.9 and 1.0 is deemed outstanding [51]. This study employed an accuracy threshold of 80% to conduct power and sample size estimations. Additionally, a slightly improved accuracy of 81% was also explored for these estimations (Table 6).

Analysis revealed that as the sample size increased, there was a corresponding rise in power and a reduction in the confidence interval range for sensitivity and specificity (Table 6). The findings suggest that a sample size of 175 participants may be necessary to achieve over 80% power in detecting a diagnostic accuracy of 81% (Table 6).

## 4. Discussion

This study revealed that predicted PPG_4–7.9h_ demonstrated a commendable diagnostic accuracy of 87.3% for identifying diabetes. At the optimal cutoff of 102.5 mg/dL, predicted PPG_4–7.9h_ exhibited a sensitivity of 75.1% and specificity of 84.1%. Utilizing simulation on 10,000 random samples, power and sample size estimations indicated that future investigations into PPG_4–7.9h_ as a diagnostic marker for diabetes may require a minimum of 175 participants.

This study demonstrated an accuracy of 87.3% (indicated by the area under the ROC curve) for predicted PPG_4–7.9h_ in diagnosing diabetes with a sensitivity of 75.1% and specificity of 84.1% at the optimal cut-off. This indicates that the capacity of PPG_4–7.9h_ for diabetes is within the excellent accuracy range of 80% to 90% [51]. This accuracy is higher than HbA_lc_. For example, it has been reported that in 2332 Chinese individuals, the diagnostic accuracy of HbA_lc_ for diabetes was 67%, with a sensitivity of about 63% and a specificity of about 62% [29]. In the Finnish Diabetes Prevention Study, HbA_lc_ of ≥6.5% diagnosed diabetes with a sensitivity of 35% in women and 47% in men [53]. Another report showed that the average sensitivity of HbA_lc_ of ≥6.5% in diagnosing diabetes among studies from six countries (Denmark, UK, Australia, Greenland, Kenya and India) was 44% [28].

In addition, the sensitivity of fasting plasma glucose of ≥126 mg/dL to detect OGTT-diagnosed diabetes was 44.7% in Japanese individuals [54]. The corresponding figure was 70.1% in UK individuals [55] and 41% in US individuals [56].

Therefore, predicted PPG_4–7.9h_ may have a better sensitivity and accuracy than HbA_lc_ and fasting plasma glucose in diabetes diagnosis. However, whether this is the case for actual PPG_4–7.9h_ needs to be investigated in the future.

PPG_4–7.9h_ displays positive correlations with mortality across various diseases, including hypertension, diabetes, cardiovascular disease, and cancer [21,22]. Notably, PPG_4–7.9h_ appears to exhibit stronger associations with certain disease outcomes compared to HbA_1c_. Specifically, the relationship between PPG_4–7.9h_ and mortality from hypertension, cardiovascular disease [22], and cancer [21] are independent of HbA_1c_. However, HbA_1c_ is not associated with cancer mortality [21] or all-cause mortality [17]. In addition, fasting plasma glucose and 2 h plasma glucose during OGTT were not associated with cancer mortality [21]. These results suggest that PPG_4–7.9h_ may be superior to the current diabetes diagnostic markers in predicting clinical outcomes.

In addition, unlike fasting plasma glucose and 2 h plasma glucose during OGTT, PPG_4–7.9h_ offers the convenience of measurement without requiring fasting, further underscoring its potential clinical utility. Moreover, the glucose test is cheaper than the HbA_1c_ test [30,31]. Consequently, validating PPG_4–7.9h_ as an additional diagnostic marker for diabetes may hold significant promise for future clinical practice.

This study found that PPG_4–7.9h_ was stable over the duration of 4 to 7.9 h, which was evidenced by the observation that fasting time did not influence its levels. This finding aligns with previous research indicating consistent hourly PPG_4–7.9h_ levels within this time frame [16,21,22]. Additionally, it echoes findings from Eichenlau et al. [20], who showed that plasma glucose returned to baseline four hours after a meal regardless of meal type and meal time, suggesting that PPG_4–7.9h_ may reflect an individual’s state of glucose homeostasis.

The optimal cutoff of 102.5 mg/dL for predicted PPG_4–7.9h_ falls below the current fasting plasma glucose cutoff for diabetes diagnosis (126 mg/dL) [23,26]. This observation is consistent with prior reports indicating lower PPG_4–7.9h_ values compared to fasting plasma glucose in individuals with diabetes under good control [57,58]. For example, Avignon et al. [57] reported that in patients with type 2 diabetes who had good diabetic control (HbA_lc_ < 7.0%), the PPG_4–7.9h_ level (measured 5 h after lunch) was 104 mg/dL while the fasting plasma glucose level in those patients was 133 mg/dL. Similarly, Peter et al. [58] reported that in patients with type 2 diabetes who had good diabetic control (HbA_lc_ < 7.3%), the PPG_4–7.9h_ level (measured 4 h after breakfast, lunch, or dinner) was 102 mg/dL while the fasting plasma glucose level in those patients was 127 mg/dL. The common observation of higher fasting plasma glucose than PPG_4–7.9h_ in those with type 2 diabetes may result from a transient increase in both glycogenolysis and gluconeogenesis in the early morning [59], a phenomenon termed “dawn phenomenon” [60].

The identified cutoff of 102.5 mg/dL for diabetes diagnosis corresponds closely to PPG_4–7.9h_ levels of 102–104 mg/dL observed in type 2 diabetes patients maintaining relatively good control [57,58]. Furthermore, this cutoff mirrors the PPG_4–7.9h_ threshold associated with cancer mortality (101 mg/dL) [21].

Strengths of the study include its relatively large sample size (*n* = 4420 for the postprandial group and *n* = 8422 for the fasting group) and the incorporation of numerous variables to estimate PPG_4–7.9h_ levels. However, a limitation lies in the use of prediction of PPG_4–7.9h_ while investigating its utility for diabetes diagnosis. Nevertheless, the predictive model, consisting of 30 predictors, performed satisfactorily, with 80% of participants having a predicted PPG_4–7.9h_ within 11.1 mg/dL of the true value. By providing insights into sample size estimation, this study enables researchers to properly design future studies aimed at elucidating the true value of PPG_4–7.9h_ in diabetes diagnosis.

## 5. Conclusions

Predicted PPG_4–7.9h_ appears to serve as a promising diagnostic indicator for diabetes. Subsequent studies seeking to ascertain its definitive diagnostic value might require a minimum sample size of 175 participants.

## Figures and Tables

**Table 1:** Baseline characteristics of participants.

Variables	Group 1 (Postprandial Group)	Group 2 (Fasting Group)
Sample size	4420	8422
PPG_4–7.9h_, mg/dL, median (IQR)	92 (87–98)	N/A
FPG, mg/dL, median (IQR)	N/A	99 (92–106)
2 h PG during OGTT, mg/dL, median (IQR)	N/A	109 (88–138)
Age, year, mean (SD)	49 (19)	48 (17)
Sex (male), *n* (%)	2042 (46)	4240 (50)
BMI, kg/m^2^, median (IQR)	26 (23–30)	28 (24–32)
Ethnicity, *n* (%)		
Non-Hispanic white	2104 (48)	4061 (48)
Non-Hispanic black	1033 (23)	1498 (18)
Hispanic	1220 (28)	2148 (26)
Other	63 (1)	715 (9)
Education, *n* (%)		
<High School	1679 (38)	1985 (24)
High School	1375 (31)	1928 (23)
>High School	1366 (31)	4509 (54)
Poverty/income ratio, *n* (%)		
<130%	1176 (27)	2367 (28)
130–349%	1822 (41)	2888 (34)
≥350%	1081 (25)	2591 (31)
Unknown	341 (8)	576 (7)
Physical activity, *n* (%)		
Active	1594 (36)	2108 (25)
Insufficiently active	1890 (43)	2464 (29)
Inactive	936 (21)	3850 (46)
Alcohol consumption, *n* (%)		
0 drinks/week	755 (17)	1379 (16)
<1 drink/week	532 (12)	2459 (29)
1–6 drinks/week	870 (20)	2032 (24)
≥7 drinks/week	578 (13)	1241 (15)
Unknown	1685 (38)	1311 (16)
Smoking status, *n* (%)		
Current smoker	1094 (25)	1773 (21)
Past smoker	1127 (26)	2018 (24)
Non-smoker	2199 (50)	4631 (55)
Dietary carbohydrate intake, g/day, median (IQR)	235 (171–313)	239 (182–311)
Dietary protein intake, g/day, median (IQR)	71 (51–97)	76 (57–99)
Dietary fat intake, g/day, median (IQR)	68 (45–101)	71 (51–97)
Dietary caloric intake, kcal/day, median (IQR)	1899 (1392–2525)	1969 (1507–2513)
SBP, mm Hg, median (IQR)	123 (112–138)	119 (110–131)
TC, mg/dL, median (IQR)	204 (177–235)	193 (167–220)
HDL-C, mg/dL, median (IQR)	50 (41–60)	52 (43–63)
Use of antihypertensive medication		
No	3384 (77)	6053 (72)
Yes	693 (16)	1916 (23)
Unknown	343 (8)	453 (5)
Use of cholesterol-lowering medication		
No	1655 (37)	4617 (55)
Yes	134 (3)	1202 (14)
Unknown	2631 (60)	2603 (31)
Cancer diagnosis, *n* (%)		
No	4029 (91)	7694 (91)
Yes	391 (9)	728 (9)
Family history of diabetes, *n* (%)		
No	2424 (55)	5178 (62)
Yes	1918 (43)	3082 (37)
Unknown	78 (2)	162 (2)
Serum potassium, mM, mean (SD)	4.0 (0.3)	4.0 (0.3)
Serum calcium, mM, mean (SD)	2.3 (0.1)	2.3 (0.1)
Serum sodium, mM, mean (SD)	141.3 (2.5)	139.4 (2.2)
Serum phosphorus, mM, mean (SD)	1.2 (0.2)	1.2 (0.2)
Serum bicarbonate, mM, mean (SD)	27.8 (3.9)	25.2 (2.2)
Serum chloride, mM, mean (SD)	104 (3)	104 (3)
ALT, U/L, median (IQR)	14 (10–20)	21 (16–29)
AST, U/L, median (IQR)	20 (17–24)	23 (20–28)
Bilirubin, μM, median (IQR)	8.6 (6.8–10.3)	12.0 (10.3–15.4)
Blood urea nitrogen, mM, median (IQR)	5.0 (3.9–6.1)	4.3 (3.6–5.4)
Creatinine, μM, median (IQR)	85 (76–102)	75 (64–88)
Uric acid, μM, mean (SD)	311 (88)	326 (82)
Serum protein, g/L, mean (SD)	74 (5)	72 (5)
Serum albumin, g/L, mean (SD)	41.8 (3.7)	42.4 (3.1)
Serum insulin, μU/mL, median (IQR)	8.5 (5.9–12.7)	9.5 (6.1–15.4)
HbA_1c_, %, median (IQR)	5.3 (5.0–5.7)	5.4 (5.2–5.7)
Fasting time, h, mean (SD)	6.6 (0.8)	12.1 (1.9)

ALT, alanine aminotransferase; AST, aspartate aminotransferase; BMI, body mass index; FPG, fasting plasma glucose; HbA_1c_, hemoglobin A_1c_; HDL-C, high-density lipoprotein cholesterol; IQR, interquartile range; N/A, not available; OGTT, oral glucose tolerance test; PG, plasma glucose; PPG_4–7.9h_, postprandial plasma glucose measured from blood taken between 4 and 7.9 h; SBP, systolic blood pressure; SD, standard deviation; TC, total cholesterol.

**Table 2:** Association of potential predictors with PPG_4–7.9h_, analyzed by simple linear regression in Group 1 of 4420 participants.

Variables	B (Coefficient)	*p* Value	Variables	B (Coefficient)	*p* Value
Age	0.002	<0.001	HDL cholesterol	−0.050	<0.001
Sex			Family diabetes history		
Male	0 (ref.)		No	0 (ref.)	
Female	−0.029	<0.001	Yes	0.003	0.50
Ethnicity			Cancer		
Non-Hispanic white	0 (ref.)		No	0 (ref.)	
Non-Hispanic black	−0.028	<0.001	Yes	0.021	0.01
Hispanic	0.022	<0.001	Antihypertension medicative		
Other	0.012	0.53	No	0 (ref.)	
Body mass index	0.098	<0.001	Yes	0.052	<0.001
Education			Cholesterol-lowering medication		
<12 years	0 (ref.)		No	0 (ref.)	
12 years	−0.039	<0.001	Yes	0.021	0.12
>12 years	−0.047	<0.001	Dietary carbohydrate intake	−0.019	<0.001
Income			Dietary protein intake	−0.007	0.07
<130%	0 (ref.)		Dietary fat intake	−0.013	<0.001
130%–349%	−0.003	0.59	Dietary caloric intake	−0.018	<0.001
≥350%	−0.015	0.02	Serum potassium	0.026	<0.001
Unknown	0.025	0.01	Serum calcium	0.157	<0.001
Physical activity			Serum sodium	−0.001	0.16
Inactive	0 (ref.)		Serum phosphorus	−0.05	<0.001
Active	−0.023	<0.001	Serum bicarbonate	0.002	0.002
Insufficiently active	−0.017	0.005	Serum chloride	−0.004	<0.001
Alcohol consumption			Alanine aminotransferase	0.028	<0.001
0 drinks/week	0 (ref.)		Aspartate aminotransferase	0.02	<0.001
<1 drink/week	−0.023	0.006	Bilirubin	0.019	<0.001
1–6 drinks/week	−0.028	<0.001	Blood urea nitrogen	0.052	<0.001
≥7 drinks/week	−0.012	0.15	Creatinine	0.026	0.01
Unknown	−0.016	0.01	Uric acid	0.0002	<0.001
Smoking status			Serum protein	0.001	0.14
Nonsmoker	0 (ref.)		Serum albumin	−0.0002	0.75
Current smoker	−0.005	0.36	Serum insulin	0.070	<0.001
Past smoker	0.024	<0.001	Hemoglobin A_1c_	0.705	<0.001
SBP	0.235	<0.001	Fasting time	−0.002	0.70
Total cholesterol	0.075	<0.001			

The following variables were natural log transformed to improve data distribution prior to simple linear regression: PPG_4–7.9h_, body mass index, systolic blood pressure, total cholesterol, HDL cholesterol, dietary carbohydrate intake, dietary protein intake, dietary fat intake, dietary caloric intake, alanine aminotransferase, aspartate aminotransferase, bilirubin, blood urea nitrogen, creatinine, serum insulin, and blood hemoglobin A_1c_. HDL, high-density lipoprotein; PPG_4–7.9h_, postprandial plasma glucose measured from blood taken between 4 and 7.9 h; Ref., reference; SBP, systolic blood pressure.

**Table 3:** Multiple linear regression model in predicting PPG_4–7.9h_ in Group 1 of 4420 participants.

Models	R Square	R Square Change	Significance of R Square Change
1	0.095	0.095	<0.001
2	0.118	0.023	<0.001
3	0.123	0.005	<0.001
4	0.14	0.017	<0.001
5	0.16	0.02	<0.001
6	0.203	0.042	<0.001
7	0.429	0.226	<0.001

Model 1: adjusted for age, sex, and ethnicity; Model 2: adjusted for all the factors in Model 1 plus body mass index, education, income, physical activity, smoking, alcohol intake, dietary carbohydrate intake, dietary fat intake, dietary caloric intake; Model 3: adjusted for all the factors in Model 2 plus systolic blood pressure, total cholesterol, HDL cholesterol, cancer diagnosis, and use of antihypertensive medication; Model 4: adjusted for all the factors in Model 3 plus circulating ionic profile (potassium, calcium, phosphorus, bicarbonate, and chloride); Model 5: adjusted for all the factors in Model 4 plus circulating enzymatic and metabolic profile (alanine aminotransferase, aspartate aminotransferase, bilirubin, blood urea nitrogen, creatinine, and uric acid); Model 6: adjusted for all the factors in Model 5 plus serum insulin; Model 7: adjusted for all the factors in Model 6 plus blood hemoglobin A_1c_.

**Table 4:** Coefficients of predictors in the PPG_4–7.9h_ predictive model in Group 1 of 4420 participants analyzed by multiple linear regression.

Variables	B (Coefficient)	Variables	B (Coefficient)
Age	0.001	Smoking status	
Sex		Nonsmoker	0 (reference)
Male	0 (reference)	Current smoker	−0.006
Female	−0.021	Past smoker	−0.003
Ethnicity		Systolic blood pressure	0.048
Non-Hispanic white	0 (reference)	Total cholesterol	−0.042
Non-Hispanic black	−0.05	HDL cholesterol	0.015
Hispanic	0.001	Cancer	
Other	−0.008	No	0 (reference)
Body mass index	−0.042	Yes	−0.012
Education		Antihypertensive medication	
<12 years	0 (reference)	No	0 (reference)
12 years	−0.003	Yes	−0.002
>12 years	−0.0001	Dietary carbohydrate intake	−0.02
Income		Dietary fat intake	0.031
<130%	0 (reference)	Dietary caloric intake	−0.017
130%–349%	0.001	Serum potassium	−0.004
≥350%	−0.009	Serum calcium	0.081
Unknown	0.005	Serum phosphorus	−0.03
Physical activity		Serum bicarbonate	0.001
Inactive	0 (reference)	Serum chloride	−0.001
Active	−0.002	Alanine aminotransferase	0.014
Insufficiently active	0.001	Aspartate aminotransferase	−0.024
Alcohol consumption		Bilirubin	0.032
0 drinks/week	0 (reference)	Blood urea nitrogen	0.004
<1 drink/week	0.001	Creatinine	−0.038
1–6 drinks/week	−0.002	Uric acid	−0.0001
≥7 drinks/week	0.015	Serum insulin	0.052
Unknown	−0.013	Hemoglobin A_1c_	0.661

The following variables were natural log transformed to improve data distribution prior to multiple linear regression: PPG_4–7.9h_, body mass index, systolic blood pressure, total cholesterol, HDL cholesterol, dietary carbohydrate intake, dietary fat intake, dietary caloric intake, alanine aminotransferase, aspartate aminotransferase, bilirubin, blood urea nitrogen, creatinine, serum insulin, and blood hemoglobin A_1c_. HDL, high-density lipoprotein; PPG_4–7.9h_, postprandial plasma glucose measured from blood taken between 4 and 7.9 h.

**Table 5:** Distribution of delta PPG_4–7.9h_ in Group 1 of 4420 participants.

Percentiles	Delta PPG_4–7.9h_ (mg/dL)
10	−11.1
20	−6.5
30	−3.8
40	−1.6
50	0.6
60	2.7
70	4.7
80	7.2
90	10.9

PPG_4–7.9h_, postprandial plasma glucose measured from blood taken between 4 and 7.9 h; delta PPG_4–7.9h_ = predicted value–actual value.

**Table 6:** Power estimation for predicted PPG_4–7.9h_ to diagnose diabetes.

Sample Size	*n* = 50	*n* = 100	*n* = 170	*n* = 175	*n* = 200	*n* = 300
Power for 80% accuracy	79.9%	84.2%	89.3%	89.9	91.2%	94.5%
Power for 81% accuracy	68.7%	77.8%	79.7%	81.0%	82.9%	87.6%
Sensitivity (95% CI)	75.2% (25.0%–100%)	75.4% (42.7%–100%)	75.0% (50.0%–94.4%)	75.4% (52.9%–94.4%)	75.1% (53.9%–93.8%)	75.1% (57.7%–90.5)
Specificity (95% CI)	84.2% (72.9%–93.6%)	84.1% (76.3%–91.2%)	84.1% (78.3%–89.6%)	84.1% (78.3%–89.4%)	84.1% (78.8%–89.1%)	84.1% (79.8%–88.2%)

Power was estimated using simulations on 10,000 random samples for each sample size. CI, confidence interval.

## Data Availability

All data in the current analysis are publicly available on the NHANES website (https://www.cdc.gov/nchs/nhanes/index.htm), accessed on 3 July 2023.
